# Optimizing response to desmopressin in patients with monosymptomatic nocturnal enuresis

**DOI:** 10.1007/s00467-016-3376-7

**Published:** 2016-04-12

**Authors:** Konstantinos Kamperis, Charlotte Van Herzeele, Soren Rittig, Johan Vande Walle

**Affiliations:** 1 Department of Pediatrics, Aarhus University Hospital, Skejby, Palle Juul-Jensens Boulevard 99, 8200 Aarhus N, Denmark; 2 Department of Pediatric Nephrology, University Hospital Ghent, Ghent, Belgium; 3 University of Ghent, Ghent, Belgium; 4 Department of Pediatric Nephrology, Safepedrug Consortium, University Hospital Ghent, Ghent, Belgium

**Keywords:** Desmopressin, Nocturnal enuresis, Best practices, Treatment outcome, Treatment resistance

## Abstract

Most patients with monosymptomatic nocturnal enuresis can be effectively treated with an enuresis alarm or antidiuretic therapy (desmopressin), depending on the pathophysiology of the condition in the individual patient. Desmopressin is first-line therapy for enuresis caused by nocturnal polyuria, an excessive urine output during the night. However, in a recent study, around one-third of patients thought to be resistant to desmopressin were subsequently treated effectively with desmopressin monotherapy in a specialist centre. The aim of this article is to review best practice in selecting patients for desmopressin treatment, as well as outline eight recommendations for maximizing the chances of treatment success in patients receiving desmopressin. The roles of formulation, dose, timing of administration, food and fluid intake, inter-individual variation in response, body weight, adherence, withdrawal strategies and combination therapies are discussed in light of the most recent research on desmopressin and enuresis. Possible reasons for suboptimal treatment response are explored and strategies to improve outcomes in patients for whom desmopressin is an appropriate therapy are presented. Through optimization of the treatment plan in primary and specialist care centres, the hope is that fewer patients with this distressing and often embarrassing condition will experience unnecessary delays in receiving appropriate care and achieving improvements.

## Introduction

According to the International Children’s Continence Society’s (ICCS) standardization of terminology [1], children with nocturnal enuresis (NE) with concomitant symptoms of lower urinary tract dysfunction differ clinically, therapeutically and in pathogenesis from children without daytime symptoms. Patients without daytime symptoms are categorized as having monosymptomatic nocturnal enuresis (MNE), and these patients are the focus of this review, although we do touch upon the potential role of desmopressin in patients with non-monosymptomatic NE (NMNE) whose daytime symptoms have been resolved.

MNE is generally a more straightforward condition than NMNE to treat using one of two treatments with level 1 evidence and grade A recommendations from the International Consultation on Incontinence: enuresis alarm and desmopressin [2, 3]. However, patients must be properly evaluated and diagnosed and therapy must be used appropriately for the treatment to be successful [4]. Data show that proper patient screening can predict treatment response, as well as failure rates [5]. Furthermore, in a recent study of enuresis patients with desmopressin resistance who had been referred to a specialist centre, one-third of children (177/539) subsequently became dry on desmopressin monotherapy under specialist care, once any daytime symptoms had been addressed [6].

We therefore highlight the importance of appropriate desmopressin usage and administration, and the failure of primary care centres to achieve optimal results with first-line treatment is of concern. For many patients, enuresis and its associated distress are unnecessarily prolonged despite being prescribed a potentially effective treatment. The aim of this review is to clarify best practice in the use and administration of desmopressin for eligible enuresis patients.

## MNE and its pathophysiology

Research has established that there are three key pathogenic mechanisms underlying MNE:

1. Nocturnal polyuria (NP)
  The ICCS definition [nocturnal urine production >130 % of expected bladder capacity (EBC) for age] [1] is a relatively arbitrary cutoff, above which nocturnal urine production is considered abnormal {EBC =[30 ×  (age in years +  1) mL]}. To some extent the EBC-based formula is supported by population data [7].
  In the context of using NP as an index for good desmopressin response, then we suggest that nocturnal diuresis of >100 % of EBC might be more appropriate for the clinician [5].
2. Reduced or abnormal bladder reservoir function at night [8, 9] (including isolated reduced nocturnal bladder capacity (B. Borg, K. Kamperis, S. Rittig, unpublished data).
3. Inability to wake in response to bladder signalling

Either (or both) of the first two issues may be present, causing a mismatch between the volume of urine produced overnight and the volume of urine the bladder can accommodate before emptying. However, for enuresis to result, rather than nocturia, there must also be an impairment of arousal from sleep with a full bladder.

Previous estimates have suggested that approximately two-thirds of children with MNE have NP [3]. The true figure, using current ICCS definitions, is likely to be lower, but it can only be determined in a completely unselected population for which data are not currently available.

It is widely accepted that nocturnal polyuria in the majority of children is related to an insufficient nocturnal increase in antidiuretic hormone, i.e., arginine vasopressin (AVP) [10, 11], causing a high diuresis rate with low osmolality overnight. Other mechanisms might be involved, especially in desmopressin-refractory patients, such as excessive evening fluid intake or, more commonly, factors such as abnormal circadian rhythm of osmotic excretion, natriuresis, excess prostaglandin production or an abnormal circadian rhythm of the glomerular filtration rate [12–14].

However, there is good evidence that the major pathogenetic factor in children with NP is decreased AVP overnight [10, 11]. Secretion of this hormone usually increases during sleep to allow a low volume and highly concentrated nocturnal urinary output. In many children with enuresis and NP, this circadian rhythm is lacking. Children with NP are most likely to benefit from desmopressin since lower nocturnal vasopressin levels have been demonstrated in a large percentage of patients [11], making substitution with desmopressin, a synthetic analogue of AVP, a rational first-line treatment for children with MNE and NP [3].

Children without NP are most likely to benefit from an enuresis alarm. The alarm’s mechanism of effect is not fully understood, but an increase in bladder storage capacities is reported with its use [15, 16]. Despite good efficacy when used appropriately and consistently, alarm treatment can present a significant burden to the family, and discontinuation rates are high [17].

## Desmopressin profile

### Formulations

Desmopressin is available as a solid tablet (0.2–0.6 mg), a rapidly melting oral lyophilisate (120–360 μg) and an intranasal spray. However, the NE indication has been withdrawn from the intranasal spray in most countries due to unpredictability of dosing and increased risk of hyponatremia [18].

### Efficacy

Desmopressin was first indicated for NE in 1982. A large body of research has demonstrated the drug’s efficacy [19–21]. Reported response rates vary (e.g. only 41 % of patients achieved ≥50 % reduction in wet nights in the study by Lottman et al. [22], but 77 % achieved >90 % reduction in the study by Onol and colleagues [17]), likely affected by the type of patients selected, suboptimal adherence rates, administration methods and doses and formulations used. In general, it is estimated that around 30 % of children with enuresis are full responders to desmopressin and that 40 % have a partial response to this AVP analogue [2].

### Safety

Desmopressin is generally well tolerated [23]. A rare but serious side effect is low serum sodium (hyponatremia); predisposing factors include excessive fluid intake, high doses, use of the intranasal spray formulation (historically) and, in particular, concomitant medications or illnesses [18, 24]. However, if used correctly, oral desmopressin has a good safety profile in children with MNE, regardless of age and gender [18, 25].

According to ICCS recommendations, an evening fluid intake of ≤200 ml and then no drinking until morning is a safe guideline to minimize risk of hyponatremia [2]. In general practice, it is commonly advised that patients should stop drinking 2 h before bedtime, with desmopressin administration up to 1 h before bedtime. Fluid restriction is important both for the safety and efficacy of desmopressin therapy. Patients and medical professionals should be vigilant to symptoms such as nausea, headache and vomiting, especially during the first 2 weeks following treatment initiation when hyponatremia is most likely [26].

## Which patients can benefit from desmopressin?

A post-hoc analysis of the large DRIP [Desmopressin Response in PNE (primary nocturnal enuresis)] study demonstrated the importance of selecting the right treatment for the right patients, based upon a full non-invasive medical evaluation, including a frequency–volume chart [5]. The original DRIP study found that around 40 % of participants experienced ≥50 % reduction in wet nights, a lower than expected response rate [22]. Entry criteria for the study excluded those with “daytime symptoms”, and all eligible subjects were considered to have MNE. However, strict ICCS criteria were not applied as the study was designed and carried out prior to publication of the 2006 standardization paper which clearly defined characteristics of patients requiring an MNE versus NMNE diagnosis.

A recent post-hoc analysis investigated parameters that were predictive of response to desmopressin in the DRIP study [5] and found that age was the only significant demographic predictor (increased efficacy with increased age). Younger children are more prone to have a low maximum voided volume (MVV) and/or overactive bladder symptoms which are resistant to antidiuretic therapy [8, 27]. The relevance of an interaction between food ingested and desmopressin tablets is also greater for younger children (see below), since they are likely to have a shorter interval between their last meal and bedtime than older children. Controlling for age, significant predictive clinical variables were number of wet nights per week (increased efficacy with fewer wet nights), average daytime voided volume, maximum daytime voided volume, total daytime diuresis, nocturnal diuresis, maximum 24 h voided volume and total 24 h diuresis. In fact, >80 % of children included in the DRIP study did not have NP (using the definition proposed by Rittig [7]) and had a low daytime MVV (using the definition proposed by Hjälmås [28]), as shown in Table 1. This indicates that desmopressin was not the most appropriate treatment for these children and helps to explain the rather low response rate. The strongest individual predictors of treatment success were increased nocturnal diuresis and fewer wet nights per week.

**Table 1:** Nocturnal polyuria and maximum voided volume status of children included in the DRIP study^a^

Nocturnal polyuria	Normal or high maximum voided volume	Low maximum voided volume^b^ i.e. below [30 + (age × 30) ]
Nocturnal polyuria^c^ i.e. nocturnal urine volume of more than [20 × (age + 9) ml ]	3.16 %	9.63 %
No nocturnal polyuria	6.98 %	80.23 %

^a^DRIP [Desmopressin Response in PNE (primary nocturnal enuresis)] study (Lottmann et al. [22])

^b^Low maximum voided volume defined using cut-off proposed by Hjälmås [28]

^c^Nocturnal polyuria (NP) is defined using cutoff proposed by Rittig et al. [7]

Previous studies have highlighted the influence of bladder capacity (voided volumes) on response to desmopressin. Patients with an MVV of >70 % of that expected for age are twice as likely to respond to desmopressin compared with patients with a reduced MVV [27]. Therefore, it is essential that the treating physician recognize that desmopressin will not work for all patients and that he/she does have some tools to predict response. It is important that the most appropriate treatment strategy is selected as quickly as possible in order to minimize distress and difficulty for the patient and family. Thorough history-taking is therefore required to identify complicating factors, such as constipation, psychological problems, daytime urinary symptoms, among others, which should be addressed first and/or which indicate a need for referral for specialist evaluation [4].

The use of bladder diaries is highly recommended wherever possible [4]. This should include a daytime diary (frequency–volume chart) documenting void volumes and times and fluid intake over a 4-day period, which is sufficient time to allow an evaluation of MVV [29]. Although the ICCS advises the exclusion of the first morning void [1], we recommend that it should be included for the MVV to be predictive of desmopressin response [5, 30]. We recognize that the ICCS standardization recommends the use of 2-day diaries, which represent a compromise between optimal and minimal registration during screening [1]. However, we advocate the longer 4-day diaries to optimize reliability since there is high intra-individual variability [29]. In addition, a bedwetting diary should be completed, documenting wet nights, urine volumes (or diaper weight) and time in bed for 7 consecutive nights; this diary enables the detection of NP. The volume of the first morning void (in ml) should be added to the difference in diaper weight to calculate nighttime urine production. In patients with nocturia, the volume of nighttime voids should be added [4]. Nocturnal urine volumes greater than the EBC are suggestive of NP. Note, however, that NP should be expected only on wet nights.

Once the results of these assessments are available, issues such as undetected daytime symptoms (NMNE) and other known predictors of treatment resistance can be identified, and suitability of the patient for desmopressin or the enuresis alarm can be gauged [4] (Table 2).

**Table 2:** First-line treatment choice based on nocturnal polyuria and maximum voided volume status

Presentation		Recommended first-line treatment	
NP on wet nights	Low MVV	Desmopressin	Alarm
✓	x	✓	
x	✓		✓
x	x	✓ or	✓
✓	✓	✓ and	✓

MVV, Maximum voided volume

The ICCS provides standardized definitions of treatment success and treatment response for research purposes. However, in the clinical scenario it is the affected child and family who decide appropriate criteria for treatment success [1], and patients and carers should be encouraged to return to clinic if they are not satisfied with the level of response achieved.

## How to maximize desmopressin success in the right patient

Taking into account pathophysiology and predictors of treatment success, the “textbook” candidate for desmopressin treatment:

- has NP and normal (or large) MVV based on diaries [27]
- may be at the older end of the affected age range [5, 27] (due to increased food interaction and prevalence of overactive bladder (OAB) in younger children)
- may have less severe NE [5]

Once patients have been identified as likely to benefit from desmopressin a number of important considerations can help to achieve clinical response and may improve response in those who appear desmopressin-resistant or partially resistant (see Table 3 for summary).

**Table 3:** Summary of important considerations to take into account for treatment success with desmopressin

Consideration	Description or recommended action
Does patient have monosymptomatic NE?	History taking, frequency–volume chart (daytime symptoms, low MVV?)
Does patient have NP?	Diagnosed using bedwetting diary
Is the most appropriate formulation of desmopressin being prescribed?	Usually oral lyophilisate (higher bioavailability, predictability, less food interaction)
Timing of desmopressin administration	Ideally to be taken 1 h before bedtime and 2 h after food; oral lyophilisate should be used if shorter interval due to reduced food interaction. Some patients may take longer to reach maximum concentrating capacity; then try earlier administration
Fluid intake	Limit fluid intake from 1 h before to 8 h after administration
Desmopressin dose—is duration of action sufficient?	Inter-individual variation in desmopressin response means dose adjustment often required (Fig. 1); pharmacodynamic testing may be helpful
Body weight	Body weight may influence required dose with oral lyophilisate
Is patient adherent?	Adherence to treatment and administration recommendations is crucial but often suboptimal
Does patient want to stop treatment?	Structured/tapered withdrawal may be tried to avoid relapse
Combination therapy	Additional therapies may help in desmopressin-resistant patients or those with partial response

*NE* nocturnal enuresis; *NP* nocturnal polyuria; *MVV* maximum voided volume

### Recommendation 1: Select the most appropriate formulation—most often the oral lyophilisate formulation

There are several practical and physiological reasons why the oral lyophilisate formulation of desmopressin is in most cases the preferred formulation for children with MNE. These include:

1. Oral lyophilisate formulations are easy for children to take, and recommended for the pediatric population [31].
2. The oral lyophilisate formulation of desmopressin is preferred to the tablet, by children <12 years [32].
3. No water is required, leading to:
  reduced diuresis
  reduced fluid intake (as recommended to minimize hyponatremia risk and increase efficacy)
  increased convenience.
  4. Low food interaction is seen with the oral lyophilisate versus tablet [33], reducing difficulties posed by the short interval between the evening meal and bedtime for young children (see Recommendation 2).

#### Efficacy

There is some evidence that the oral lyophilisate may, despite bioequivalent dosing, achieve a greater reduction in the number of wet nights than the tablet [34]. This may be a result of the different pharmacokinetic and pharmacodynamic characteristics of the oral lyophilisate, administration method and/or effects of the formulation on adherence (discussed later in this review).

#### Bioavailability and predictability of dosing

 The oral lyophilisate’s higher bioavailability compared with the tablet allows lower dosing for optimal efficacy (thereby reducing the risk of side effects) [35]. It has been demonstrated that nocturnal urine production during desmopressin treatment is significantly greater during wet nights than during dry nights [36, 37], indicating nightly intra-individual variation in the antidiuretic effect (and therefore anti-enuretic effect) of desmopressin. The pharmacokinetics of desmopressin oral lyophilisate are more predictable than those of the tablet formulation, with smaller variances in plasma concentration [38], which may lead to reduced intra-individual variability and greater consistency of the antidiuretic effect.

### Recommendation 2: Ensure optimal timing of administration and consider possible impact of meals

The desmopressin tablet and oral lyophilisate should be administered 60 min before bedtime [2, 35]. However, in practice, many patients take desmopressin at bedtime. The clinical relevance of this common non-adherence to recommendations has not been fully explored. and future studies aimed at examining any impact on the drug’s efficacy would be helpful to clarify the importance of a 1-h interval between desmopressin administration and bedtime.

 Additionally, patients are advised to take desmopressin at least 2 h after the evening meal. If the drug is to be taken 1 h before bedtime, there must be a 3-h interval overall between the evening meal and bedtime, which may be impractical in school-aged children. The timing of medication administration is therefore likely, in reality, to fall short of recommended best practice, with food and desmopressin intake occurring at a similar time. Pharmacodynamic data show that the oral lyophilisate (120 μg) may be more effective and predictable than the bioequivalent 0.2 mg tablet when administered after a meal [33], with a significant increase in duration of action and indications of a shorter time to reach maximal antidiuresis and a higher concentrating capacity. Variability (standard deviation) in the diuresis rate was also lower with the oral lyophilisate, likely due to less interference with nutrition since the oral lyophilisate is believed to be reabsorbed by oral and/or oesophageal mucosa. Although to date no studies have proven this difference to be relevant for the clinical effect of the formulations, it may be an important factor for young children because of their early bedtime.

Furthermore, because the oral lyophilisate is believed to be absorbed by oral and/or oesophageal mucosa, it is likely to be less affected by intestinal motility. However, the absorption of desmopressin tablets increases if intestinal motility is delayed [39].

 Additional consideration should be given to the possible impact of diet and daytime fluid intake on osmotic load and NE. Some children with desmopressin-resistant NP have high osmotic excretion during the night, possibly due to a high osmotic load of protein and sodium during the evening meal [12]. Other children with NP may have high osmotic excretion at night but deficient osmotic excretion during the day, possibly caused by extremely low daytime fluid intake to compensate for a small bladder capacity. Adaptations to nutritional and fluid intake may therefore be helpful in improving NE in some cases.

### Recommendation 3: Ensure fluid restriction before and after desmopressin administration

Suboptimal response to desmopressin can also be worsened by failure to restrict fluid intake before the administration of desmopressin [40]. Clear instructions should be given to patients regarding fluid restriction (for efficacy and safety reasons) from 1 h before desmopressin is administered and for 8 h subsequently to encourage optimal concentrating capacity and treatment response, as well as to reduce the risk of hyponatremia/water intoxication. Patients should also be sure to go to the toilet for their final void of the day immediately before going to sleep.

### Recommendation 4: If necessary, tailor treatment dose and timing to the response of the individual

**Fig. 1:**
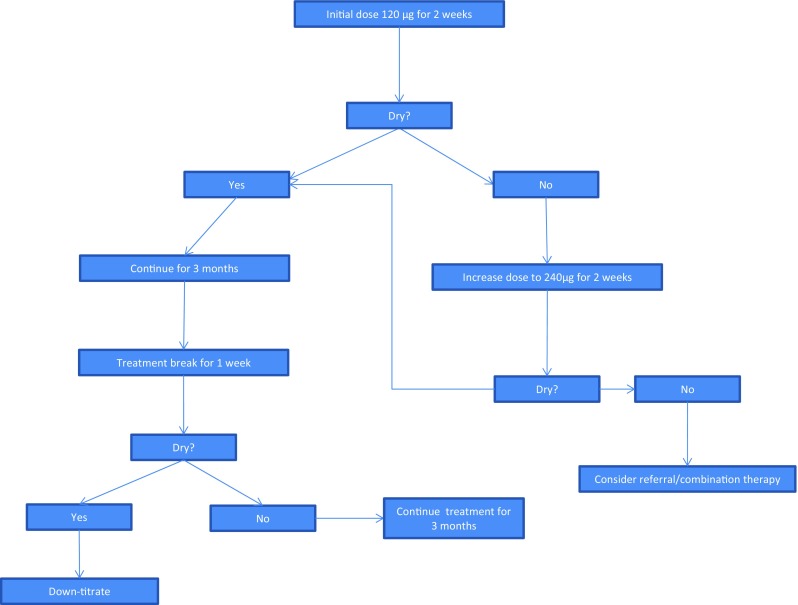
Dosing with desmopressin oral lyophilisate

The dose–response effect of desmopressin is seen not only in the level of antidiuresis achieved, but primarily in the duration of action [23]. Even at low doses, maximal antidiuresis is demonstrated, but the duration of this activity is dependent on the exposure to desmopressin, i.e. overdosing results in prolonged activity [41]. For children with MNE, the clinically relevant period is 7–11 h, i.e. equivalent to a night’s sleep. A duration of action exceeding the normal duration of a night will increase the risk of undesired prolonged antidiuresis. Pharmacodynamic data available for the lyophilisate formulation indicate that a small dose range (120–240 μg) is likely to control diuresis for a period corresponding to a night’s sleep (mean of 7–11 h) in most children with PNE [35]. A flow-chart which can be used to guide dose adjustments when prescribing the oral lyophilisate is shown in Fig. 1.

However, the inter-individual range of duration of activity is large, and an individual approach may be needed. Registry data from Denmark on desmopressin prescriptions for NE show that among 40,596 patients, 66 % used the oral lyophilisate, 18 % used tablets and 17 % used the nasal spray. Among those using the lyophilisate, 26 % used 60 μg—i.e. below the recommended minimum dose in Denmark (M. Schroeder, K, Juul, J.P. Norgaard, S. Rittig, unpublished data), suggesting this dose was sufficient for many patients. Indeed, patients with NE and NP can differ in their response to desmopressin. Time to reach maximal antidiuretic effect and the duration of pharmacodynamic action show a wide range, and there is inter-individual variation in the duration of effect [40]. Individualized tailoring and titration of dose (and hence duration of action) may therefore help to achieve efficacy in partially resistant patients [40]. A simple pharmacodynamic test based on home recordings may provide important information on optimal time of dosing, duration of action and influence of oral fluid intake, thereby allowing optimization of therapy. Due to the documented prolonged action of desmopressin in some patients, pharmacodynamic testing is required before the dose is increased above recommended levels; such testing should only be considered in specialist centres.

As discussed in previous sections, data show that desmopressin should be administered at least 1 h before bedtime to achieve optimal efficacy during sleep [35, 40]. In cases of therapy resistance, a longer interval between administration and bedtime (up to 2 h) might further reduce the diuresis rate in the early night for children who take longer to reach maximal concentrating capacity [40]. In support of this proposal, time to reach maximum antidiuretic action was found to be around 2 h in a group of children with MNE and NP who had inadequate response to desmopressin intranasal spray [40]. However, no controlled studies on increasing the interval between drug administration and bedtime have been performed, and it is possible that earlier dosing would also lead to loss of therapeutic effect before it is time for the child to rise in the morning.

 In some cases, poor treatment response due to an insufficient pharmacodynamic effect of desmopressin may be related to an inappropriate renal response with suboptimal maximal renal concentrating capacity [40], and factors such as large osmotic load, natriuresis and hypercalcaemia may play a role [14, 42]; an individual approach is recommended in these patients.

### Recommendation 5: Consider the possible impact of body weight

There is a positive correlation between the plasma concentration of desmopressin and the dose corrected by weight at 2 and 6 h post-dosing using the oral lyophilisate in children, but this weight-dependency is not seen for the tablet (nor for the intranasal spray) [38]. It has been suggested that the lack of evidence for a size effect with the administration of the tablet and the intranasal spray should be attributed to the poor predictability of their bioavailability, which may mask the size effect. If using the oral lyophilisate, therefore, it is possible that dose may need to be adapted to body weight if initial efficacy is suboptimal (see Recommendation 4).

### Recommendation 6. Ensure patients are adherent to treatment and administration recommendations

Desmopressin is only effective on the night following its administration and, consequently high levels of adherence are necessary to maintain a good response each and every night. However, in almost every therapy area, and particularly chronic conditions, adherence levels are suboptimal; around 50 % of patients do not take their medications as prescribed [43]. In a report on adherence, the World Health Organization quoted a statement by Haynes et al. that: “increasing the effectiveness of adherence interventions may have a far greater impact on the health of the population than any improvement in specific medical treatments.”

 In the DRIP trial, where desmopressin tablets were used, 81–91 % of patients ingested all medication as instructed during the initial run-in [44]. This decreased to 77 and 71 % during the first and second 3-month treatment periods, respectively. Despite the closely monitored setting of a clinical trial, therefore, up to around 20 % of patients were not fully adherent to treatment, even during the run-in phase, and over 30 % were not fully adherent at later stages. Poor adherence to treatment could therefore explain a poor response in some patients since the reduction in wet nights/week, as would be expected, is significantly greater for a higher adherence rate compared with lower adherence [34, 44]. It is crucial that clinicians advise patients of the importance of full adherence with desmopressin therapy, as well as adherence with the instructions regarding the timings of desmopressin administration, fluid intake, among other factors (discussed earlier in this review) to achieve the best possible efficacy. Ongoing clinical support and monitoring may help to facilitate this.

 There are some data to suggest that adherence to treatment increases when patients are switched from the tablet to the oral lyophilisate formulation of desmopressin [34]. This, coupled with younger children’s preference for the lyophilisate [32], suggests it may contribute to improved outcomes via higher adherence levels.

### Recommendation 7. If cessation of treatment is desired, consider a structured withdrawal programme

Following achievement of response to desmopressin, it is generally considered that continued treatment is required to avoid relapse, given that the drug only has an effect on the night following its administration for approximately 7–11 h. Regular drug holidays are recommended to evaluate whether treatment can be discontinued. There have been a small number of studies reporting that a gradual or tapered withdrawal of desmopressin over several weeks can reduce relapse compared with abrupt termination of therapy in patients using the lyophilisate [45, 46] and the tablet [47]. Other studies have failed to replicate this effect [48], and the mechanism of continued antidiuretic effect after withdrawal of treatment is not yet understood.

### Recommendation 8: Consider combination therapy where appropriate

It is possible that, in some patients, desmopressin has an antidiuretic effect but not an anti-enuretic effect—i.e. that despite reduced diuresis, bladder dysfunction still causes the bladder to empty prematurely (and patients do not wake in response to bladder signalling). Some patients have an isolated low bladder capacity which is limited to the nighttime and would not therefore present with daytime urinary symptoms or be identified as having NMNE (B. Borg, K. Kamperis, S. Rittig, unpublished data). In such patients, home recordings are recommended while the patient is receiving desmopressin to investigate how treatment may be optimized. Accurate differentiation between inadequate antidiuretic versus anti-enuretic response to desmopressin can help inform subsequent treatment choices. For some patients with confirmed MNE and NP, combination therapy may be appropriate to achieve a good response (Table 4). Such combinations should be considered only in specialist centres, with the exception of desmopressin combined with an enuresis alarm which can be used in primary care.

**Table 4:** Combination therapy options with desmopressin

Desmopressin plus:	For patients with:
Enuresis alarm	NP and small nocturnal MVV (Kamperis et al. [50]
Anticholinergic	NP and small MVV due to detrusor overactivity during sleep
Non-steroidal anti-inflammatory drug	NP and high prostaglandin levels (Kamperis et al. [51])
Diuretics during daytime	NP and abnormal circadian rhythm of renal tubular sodium handling (De Guchtenaere et al. [49])

*NP* nocturnal polyuria; *MVV* maximum voided volume

## Persistent lack of response

For some patients, despite optimization of the desmopressin regimen as described, there may still be a lack of effect. The reasons for no response or partial response include various factors. For example, the antidiuretic effect may be suboptimal, perhaps due to renal factors or an individual need for higher dosing. Desmopressin may have been inappropriately selected, as when the child has daytime urinary symptoms, especially as these may be masked by reduced daytime fluid intake. In this case, alternative treatment should be sought. Poor adherence to treatment or to administration recommendations (e.g. timing, fluid intake) may lead to poor effect, even though desmopressin is an appropriate therapy. Alternatively, if the patient has low nocturnal bladder capacity, alternative or additional treatments may be needed.

## Conclusions

In most cases, MNE can be treated successfully in primary care with an enuresis alarm or desmopressin. For treatment with desmopressin to be successful, the patient should fulfil the characteristics required for response (i.e. NP, and no NMNE) and be fully adherent to treatment and recommendations regarding the administration and food/fluid intake.

A surprisingly high proportion of patients who are believed to be initially resistant to desmopressin are, in fact, treated successfully with desmopressin monotherapy under specialist care. In this review, we have outlined eight key recommendations for the optimization of the desmopressin regimen to achieve the most successful outcome for each patient receiving the drug. The oral lyophilisate formulation is recommended for initial treatment due to several pharmacodynamic, pharmacokinetic and practical characteristics which render it superior to the tablet formulation, such as bioavailability, reduced variability, reduced interference from food, administration without water and increased adherence. However, there may be individual reasons (e.g. cost, preference) which would favour starting with, or switching to, another formulation. Incorporation of the recommendations included in this review into standard clinical practice should help to improve response to desmopressin for patients where it is the appropriate treatment; continued poor response despite following these recommendations indicates the need to explore alternative therapeutic strategies.
